# Novel bioreactor internals for the cultivation of spore‐forming fungi in pellet form

**DOI:** 10.1002/elsc.202100094

**Published:** 2022-05-18

**Authors:** Winda Soerjawinata, Isabelle Kockler, Lars Wommer, Robert Frank, Anja Schüffler, Tanja Schirmeister, Roland Ulber, Percy Kampeis

**Affiliations:** ^1^ Institute for Biotechnical Process Design Trier University of Applied Sciences, Environmental Campus Birkenfeld Hoppstädten‐Weiersbach Germany; ^2^ Institut für Biotechnologie und Wirkstoff‐Forschung gGmbH (IBWF) Mainz Germany; ^3^ Institute of Pharmaceutical and Biomedical Sciences Johannes Gutenberg University of Mainz Mainz Germany; ^4^ Institute of Bioprocess Engineering Technical University Kaiserslautern Kaiserslautern Germany

**Keywords:** bioreactor internals, fungal growth, fungal pellets, repeated‐batch fermentation, sporulation conditions

## Abstract

This study introduced an automated long‐term fermentation process for fungals grown in pellet form. The goal was to reduce the overgrowth of bioreactor internals and sensors while better rheological properties in the fermentation broth, such as oxygen transfer and mixing time, can be achieved. Because this could not be accomplished with continuous culture and fed‐batch fermentation, repeated‐batch fermentation was implemented with the help of additional bioreactor internals (“sporulation supports”). This should capture some biomass during fermentation. After harvesting the suspended biomass, intermediate cleaning was performed using a cleaning device. The biomass retained on the sporulation support went through the sporulation phase. The spores were subsequently used as inocula for the next batch. The reason for this approach was that the retained pellets could otherwise cause problems (*e.g*., overgrowth on sensors) in subsequent batches because the fungus would then show undesirable hyphal growth. Various sporulation supports were tested for sufficient biomass fixation to start the next batch. A reproducible spore concentration within the range of the requirements could be achieved by adjusting the sporulation support (design and construction material), and an intermediate cleaning adapted to this.

**Abbreviations**
BDM: bio dry mass
CAD: computer‐aided design
Glu: glucose
HPLC: high‐performance liquid chromatography
IBWF: Institut für Biotechnologie und Wirkstoff‐Forschung gGmbH
YMG: yeast extract, malt extract, glucose

## 1 INTRODUCTION

Many fungi produce secondary metabolites that could be of pharmaceutical interest, such as antibiotics (penicillin and cephalosporins) [1], vitamins (riboflavin and β‐carotene) [2, 3], and antifungals (griseofulvin and echinocandin) [4, 5]. Another interesting bioactive metabolite is the protease‐inhibiting substance produced by the aerobic filamentous fungus *Penicillium* sp. (IBWF 040‐09), provided by the *Institut für Biotechnologie und Wirkstoff–Forschung gGmbH* (IBWF). This substance could potentially be used against pathogens, such as cysteine endopeptidases, offering the possibility of curing diseases, including trypanosomiasis — African sleeping sickness [6, 7].

In general, there are two ways to cultivate filamentous fungi, *i.e*., loose mycelia or pellets [8, 9, 10]. It has been mentioned in many studies [11, 12, 13, 14, 15, 16] that fermentation in pellet form resulted in lower viscosity of the broth in comparison to the mycelial form. Gbewonyo et al. [17] reported that the viscosity of fungal pellet fermentation was over 50 % lower than that of mycelial fermentation. The lower viscosity of the fermentation broth led to a shorter mixing time and better oxygen transfer. In addition, fungal overgrowth on bioreactor internals and sensors is lower when fungi grow as pellets.

A disadvantage to pellet morphology could be mass transport in the pellets, affecting productivity [18]. However, this should not be a serious problem in the protease inhibitor produced by *Penicillium* sp. (IBWF 040‐09) because this substance is produced as a secondary metabolite by undersupplied fungal cells [7]. Undersupply does not mean that the substrates in the medium must be completely consumed. A limited mass transfer of the substrate through the fungal pellets also leads to an undersupply of the fungus inside the pellets. Posch et al. [19] reported that pellets will continue to grow until a few hours before substrate exhaustion is reached. At this point, even though the growth of the outer pellets continues, the core pellets will stop growing. Furthermore, Meyer et al. [16] also highlighted that limited oxygen diffusion could destroy the internal pellet structure. The inhibitory activity test, with samples from fermentation with both pellets and mycelia of *Penicillium* sp. (IBWF 040‐09), showed that fermentation in the mycelial form resulted in only 24 % inhibitory activity of the cumulated rows A, B, and C in the microtiter plate (as described in [7]) in comparison to the same mass of fungus in pellet form. This indicated that mass transport limitation leading to undersupplied fungus inside the pellets is preferred for producing the target substance in this case. Because of the advantages of fermenting the fungus in pellet form regarding process engineering aspects and yield of the target substance, this growth form was the subject of the work presented here.

**PRACTICAL APPLICATION**

In long‐term fermentation with filamentous fungi, problems often arise because of the overgrowth of fungal hyphae on the internal surfaces of the bioreactor or the sensors. With sensors overgrown with hyphae, it is no longer possible to obtain reliable measurement data. Such effects are less pronounced when the fungus grows in the form of pellets. In addition to the improve rheological properties of the fermentation broth, the pellet form may also be favorable for product formation in some cases. Under such circumstances, as in the case of *Penicillium* sp. (IBWF 040‐09), repeated‐batch fermentation was considered to ensure growth in pellet form and to achieve the desired productivity. In the developed process, growth in pellet form was ensured by two measures, first, by intermediate cleaning that was possible in this process, and second, by initiating each batch phase with fresh spores instead of the retained biomass.

Protease inhibitors are expressed intracellularly by *Penicillium* sp. (IBWF 040‐09) [7]. Because an increase in intracellular products correlates with an increase in biomass yield, the process engineering concept attempts to achieve the highest possible space‐time yield of biomass at low‐operating costs. From this perspective, continuous culture is preferred. However, with filamentous fungi in continuous culture, a problem arises over time, which also occurs with pellet‐like growth: the colonization of bioreactor internals (such as aeration equipment and baffle plates) and the sensors with the fungus increases. Fungal growth in pellet form is suppressed after reaching a certain degree of “biofouling” on the bioreactor internals. The fungus then grows predominantly only in the undesired mycelial form, which consequently worsens the biofouling of the bioreactor internals. The same problem occurs with long‐lasting fed‐batch fermentation.

Based on the observations in preliminary studies, longer fermentation time causes more fragile pellets, which is also in agreement with the study on the age effect of pellets by Cronenberg et al. [20]. Consequently, they break apart more easily in the following batch, which leads to undesirable fungal growth in the mycelial form. The change in morphology after extended cultivation time has already been observed in various filamentous fungi fermentations when, for example, nutrients are limited [21]. Thereby, hyphal breakage/fragmentation and vacuolization of the older cells in hyphae can lead to autolysis [22, 23], leading to an increase in the biofouling of sensors and other internals. Cronenberg et al. [20] also reported morphological changes in the pellets of *Penicillium chrysogenum* during fermentation, from compact into fluffy pellets. This phenomenon was also shown by the change in mass transport, as oxygen transport into the core of the fluffy pellets increased again at the later stage of fermentation. However, it is most likely that oxygen was not used for metabolic activity because no glucose consumption was detected in the deeper region of the pellets during this period.

Therefore, this study reveals repeated‐batch fermentation as an alternative approach to overcome the previously described shortcomings. Compared to batch and fed‐batch fermentation, the time‐consuming and labor‐intensive production of the inoculum was eliminated. The repeated‐batch fermentation described in this present study is distinct from the procedure presented in many conventional studies, in which the biomass itself is the starting point for new biomass growth [24, 25, 26]. In the newly suggested repeated‐batch approach, a defined amount of biomass will be retained and then undergo the sporulation phase, resulting in conidia used as inoculum for the next batch fermentation. Thus, special bioreactor internals, referred to in the following as sporulation supports, were designed and tested. Two important criteria for sporulation support are that the spores resulting from the sporulation support should be sufficient to start the next batch, and fermentation should result in pellet form. To the best of our knowledge, this is the first study about repeated‐batch fermentation with the help of sporulation supports.

Furthermore, automation should be considered in the laboratory to improve the production processes. Turbidity sensors, which operate on a transmittance principle, are commonly used to monitor the growth of microorganisms. However, it is not suitable for microorganisms cultivated in pellet form because of the uneven cell distribution in the fermentation medium. Therefore, a time‐consuming and labor‐intensive offline gravimetric method has been the only available method to date. However, this method has several disadvantages, including relatively large sample volumes and non‐representative samples. To overcome these disadvantages, the suitability of a sensor based on the principle of backscattered light was examined.

When the first batch fermentation with sporulation support was completed, the biomass was harvested, and some biomass was retained on the sporulation support. Before the retained biomass underwent the sporulation phase, the bioreactor was cleaned using a self‐rotating nozzle designed, constructed, and tested in this study. This intermediate cleaning step was performed between batch phases.

## 2 MATERIALS AND METHODS

### 2.1 Design and manufacturing of the sporulation supports

The sporulation supports were designed using computer‐aided design (CAD) with the software Siemens NX 1859. Manufacturing was performed with a Vida 3D printer (EnvisionTEC GmbH) using the principle of inverse digital light processing (inverse DLP) at a temperature of 23°C. The pixel width of the light projector with a power of 330 W was 73 × 73 μm at a resolution of 1920 × 1080. The printing process was performed with a layer height of 50 μm. For this purpose, the components created in the CAD software were divided into individual layers using Perfactory Rapid Prototyping 3.2.3377.1712 software. The resins used were HTM 140 V.2 (EnvisionTEC GmbH) and E‐Shell 600 clear (DeltaMed GmbH). E‐Shell 600 clear is certified according to the United States Pharmacopeia (USP) as Class VI and thus is biocompatible according to ISO 10993, making it suitable for pharmaceutical applications.

### 2.2 Strain and inoculum preparation

The microorganisms used in this study were *Penicillium* sp. (IBWF 040‐09), which were maintained on YMG‐agar plates (9 g/L glucose, 10 g/L malt extract, 4 g/L yeast extract, 20 g/L agar, and adjusted to pH  =  5.5 using 1 M HCl). The agar plates were incubated at 22°C in an incubator (INCU‐Line, IL 23R, VWR) for 4 weeks before fermentation. Preservation of the fungi was performed using the streak plate method from 2‐week‐old spore plates to new agar plates every week. Inoculum preparation for fermentation was conducted by washing the spores from an agar plate using sterile 0.9 % NaCl solution containing 10 μL/L Triton‐X‐100. Triton‐X‐100 was used to reduce surface tension because the spores were hydrophobic.

### 2.3 Fermentations in the bioreactor and shake flasks

A glass bioreactor UniVessel Glass DW 2 L from Sartorius Stedim Biotech GmbH, equipped with two pieces of a three‐blade segment impeller (d_i_ = 54 mm; d_i_/d_v_ = 0.41), was used. Other properties of the bioreactor are explained elsewhere [7]. The fermentation medium used in this study was the YMG medium, with a composition similar to that described in Section 2.2 but without agar. A volume of 2 L of the medium was used in all experiments with the addition of 1 g/L CaCl_2_ [27, 28, 29]. Polypropylene glycol was added before sterilization and used as an antifoaming agent at a concentration of 25 μL/L of the medium. For fermentation, the medium was autoclaved in the glass bioreactor at 121°C for 15 min. *Penicillium* sp. (IBWF 040‐09) fermentation was conducted at 22°C. Before inoculation with 6.62 × 10^5^ spores/L of fermentation medium, sterile‐filtered Pluronic F68 with a final concentration of 0.2 % (v/v) was added to the medium to reduce the shear stress [30]. The initial aeration of the fermentation was set to 1 L/min (0.5 vvm). Aeration was controlled to maintain a minimum dissolved oxygen (DO) saturation of 30 % by increasing the atmospheric airflow rate to a maximum of 2.25 L/min and adding additional pure oxygen up to 0.25 L/min, as needed. The pH value during fermentation was only measured but not controlled to observe the metabolic activity of the fungus. The stirrer speed remained constant at 350 min^–1^ (refers to u_Tip_  =  1 m/s). Under these fermentation conditions, the submerged fermentation of *Penicillium* sp. (IBWF 040‐09) resulted in pellets with diameters between 2 and 4 mm. Each fermentation was terminated when the aeration rate decreased and returned to 1 L/min. During fermentation, samples were taken regularly to analyze further bio dry mass (BDM) and substrate concentration (glucose and maltose). Substrate analysis via high‐performance liquid chromatography (HPLC) was performed as previously described [7] with an additional pre‐column (Repromer Ca^2+^, 9 μm, 20 × 8 mm, Dr. Maisch GmbH). Online measurements of fungal growth were performed using the CGQ BioR (Aquila Biolabs GmbH and Infors AG).

Additional shaking flask experiments were performed in 250 mL baffled shaking flasks with a medium volume of 50 mL. The medium used was YMG medium, as described above. The shaking flask experiments were performed at 22°C with shaking at 100 min^–1^ in an incubator (HAT Ecotron, Infors AG).

### 2.4 Sporulation experiments

Pellets from shaking flask cultures were transferred into sterile Schott bottles for sporulation experiments. Three aeration conditions were used. One condition was performed without an air supply in sealed Schott bottles. The Schott bottles for the other two aeration conditions were connected to an air supply via a sterile filter, with an exhaust hose equipped with a sterile filter. In one case, the Schott bottles were aerated with dry air; in the other, the air was humidified with an upstream wash bottle. The Schott bottles were incubated in a water bath (K12‐NR, Peter Huber Kältemaschinenbau GmbH) or an incubator (Certomat HK, Sartorius AG) to maintain a constant temperature of 10°C, 16°C, 22°C, 28°C, and 37°C. Sporulation was determined after 7 days by suspending spores with 50 mL of sterile 0.9 % NaCl solution, to which 10 μL/L Triton‐X‐100 was added. The concentration of this suspension was determined using a Brightfield Cell Counter (Celldrop BF, DeNovix Inc.). A volume of 10 μL of spore suspension was used to quantify spore concentration. The focus was adjusted according to the manufacturer's instructions for every measurement.

For the sporulation experiments in the bioreactor, the entire medium, including the biomass, was pumped out of the bioreactor at the end of fermentation. To ensure reproducibility of the experiments, all biomass deposits in the bioreactor were removed in a biological safety cabinet. Only the biomass on the sporulation support remained in the bioreactor. Subsequently, aeration with humid air was set to 0.2 L/min, and the temperature control in the reactor was set to 22°C. The sporulation phase was terminated after 7 days by suspending the spores with 2 L of sterile 0.9 % NaCl solution to which 10 μL/L Triton‐X‐100 was added. The concentration of this suspension was determined analogously to the sporulation experiments in Schott bottles using a Brightfield Cell Counter (Celldrop BF, DeNovix Inc.).

### 2.5 Determination of BDM

The BDM of the cells, expressed in grams of dry weight per liter of culture medium (g/L), was determined gravimetrically. Samples (5 mL) collected during fermentation were filtered using a Büchner funnel with a predried and preweighted 0.45 μm membrane filter (ø 47 mm, PES Supor Membrane Disc Filter, PALL Life Science). The pellets were then washed with distilled water. The filter cakes were dried at 80°C in an oven (UT12P, Thermo Electron LED GmbH) until they reached a constant weight. As previously described, the statistical assessment (*t*‐test) of the correlation between BDM and backscatter was performed [7].

## 3 RESULTS AND DISCUSSION

### 3.1 Sporulation conditions

The sporulation phase occurs between the individual batch phases of the repeated‐batch process. The spores produced are used as inocula for the subsequent batch. Thus, it is important to provide optimal sporulation conditions. Two parameters must be considered, namely temperature and aeration conditions. Sporulation experiments were conducted at different temperatures (10°C, 16°C, 22°C, 28°C, and 37°C) and aeration conditions (humid aeration, dry aeration, and no aeration) in Schott flasks. Table 1 shows the spore concentrations achieved as described in Section 2.4 under different temperatures and humidity conditions.

**TABLE 1:** Sporulation in Schott bottles under different temperatures and aeration conditions after 7 d

	Temperature				
	10°C	16°C	22°C	28°C	37°C
Dry aeration	Not determinable	Not determinable	Not determinable	Not determinable	Not determinable
Humid aeration	3.16 × 10^9^ spores/L	3.43 × 10^9^ spores/L	1.22 × 10^11^ spores/L	2 × 10^8^ spores/L	Not determinable
No aeration	3.24 × 10^9^ spores/L	4.75 × 10^10^ spores/L	2.02 × 10^11^ spores/L	6.3 × 10^8^ spores/L	Not determinable

At 22°C with humid aeration, sporulation began after 3 days. At lower temperatures, sporulation occurred to a lesser extent and was visible by eye only after 5–6 days. The extent of sporulation was lower at higher temperatures. At 37°C, no sporulation was observed. These results indicated that temperature is a crucial factor. Therefore, the bioreactor should be fixed at 22°C during the sporulation phase because this temperature is also used for preparation of strain and inoculum preparation as well as fermentation [7]. As shown in Table 1, sporulation also occurred on the wet biomass without aeration because of sufficient oxygen content and humidity in the (almost empty) Schott bottles. However, it was observed that at every temperature, dry aeration caused the fungus to dry quickly before sporulation could begin. Thus, these results indicated that sufficient moisture must be available in the bioreactor, and aeration of the bioreactor with dry air should be avoided during the sporulation phase. To ensure that sufficient moisture was available during the sporulation phase, humid aeration at 22°C was used in the following bioreactor experiments.

### 3.2 Sporulation supports

Bioreactor internals, which we call sporulation supports, were developed to retain a defined amount of biomass. The geometries of these sporulation supports were designed using CAD software. The design should ensure that the biomass adhering to the sporulation supports is not detached in turbulent stirring flow or during intermediate cleaning. The retained biomass should result in a spore concentration between 6.62 × 10^5^ spores/L and 6.62 × 10^8^ spores/L after the sporulation phase, which was considered sufficient for growth in pellet form in preliminary studies. If the sporulation supports retain too much biomass, they will form too many spores, and growth will no longer occur in pellet form. If too little biomass is retained during intermediate cleaning, the spore concentration will be too low, and the production process will be undesirably slowed in the next batch phase. When optimizing the sporulation support, care must be taken to produce a reproducible spore concentration in the desired range. However, this only works if intermediate cleaning is performed accordingly. 3D printing, as described in Section 2.1, was chosen to produce the sporulation supports because this technique allows easy revision of the geometry within optimization. Two materials were tested as construction materials: E‐Shell 600 clear (DeltaMed GmbH) and HTM 140 V.2 (EnvisionTEC GmbH).

#### 3.2.1 Fixed sporulation support

**FIGURE 1:**
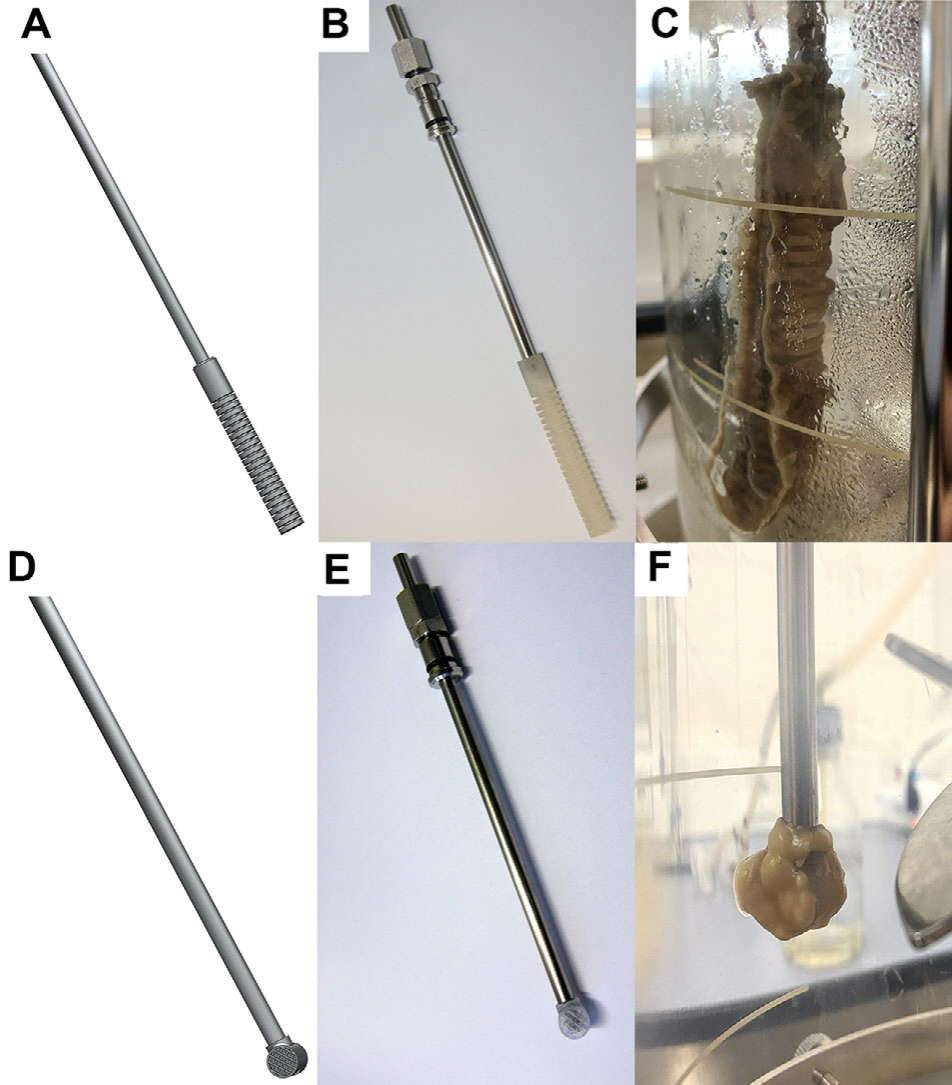
Type F1 (top row) and type F2 (bottom row) of the fixed sporulation support; CAD drawings (A and D, respectively), photos of manufactured fixed sporulation supports with E‐Shell 600 clear (B and E, respectively) and biomass on them after fermentation (C and F, respectively)

Fixed sporulation support was developed in the form of a probe. It has been designed to be installed in a common 11 mm port in the head plate of many laboratory fermenters via a 6 mm adapter. The installation height could be adjusted using a compression fitting. The distance between the sporulation support and the stirrer shaft was 49 mm. Figure 1 shows the CAD drawings of two different geometries (F1 and F2) of the fixed sporulation support and photos of the manufactured fixed sporulation supports. Figure 1C and 1F show the accumulated biomass onto the sporulation support types, F1 and F2, respectively. It can be seen that both fixed sporulation supports performed very well. The sporulation support type F1 was designed as a cylindrical body with grooves. These grooves protect the fungi from shear stress; thus, the fungi could attach and grow there. The BDM at the end of the fermentation with sporulation support type F1 (first batch phase) was 5.79 ± 0.29 g/L.

With the optimal sporulation conditions described in Section 3.1, a spore concentration of 2.25 × 10^8^ spores/L, according to Section 2.4, could be measured using the sporulation support type F1 (see Figure 1, upper row). This spore concentration is in the range of the required spore concentration, as mentioned in Section 3.2. However, it was approximately 300 times higher than the desired initial inoculum concentration of ca. 7 × 10^5^ spores/L. Therefore, further optimization was performed to reduce biomass accumulation and the subsequent sporulation of biomass. The length of the sporulation support was decreased from 80 to 10 mm, and different shapes were designed to control the growth on the sporulation support. This geometry optimization resulted in sporulation support type F2, which possesses a cylindrical hollow body structure formed by a 3D grid (see Figure 1, bottom row). The sporulation support type F2 produced better results in biomass accumulation and subsequent spore concentration achieved (3.76 × 10^7^ spores/L). This spore concentration was not as high as type F1 and was a better starting point for cultivation. A drawback was the lower BDM (4.35 ± 0.26 g/L) obtained at the end of fermentation (first batch phase).

The pH profiles of the subsequent fermentation after sporulation (second batch phase) exhibited the typical profile of fermentation with *Penicillium* sp. (IBWF 040‐09) in all experiments, as previously shown [7]. This suggests that the metabolic activity of the fungus in the experiments corresponded to fermentation inoculated with a spore suspension.

Unfortunately, during the experiments, the fixed sporulation support resulted in more free mycelia and fewer pellets compared to conventional batch fermentations with a spore suspension as the inoculum. The additional reactor installation led to higher shear forces in the fermentation broth, which destroyed the pellets. Revising the design of the fixed sporulation support (from type F1 to type F2, as described above) reduced the additional shear forces, ensuring growth mainly in pellet form. Nevertheless, other solutions were still sought (see Section 3.2.2).

#### 3.2.2 Spinning sporulation support

**FIGURE 2:**
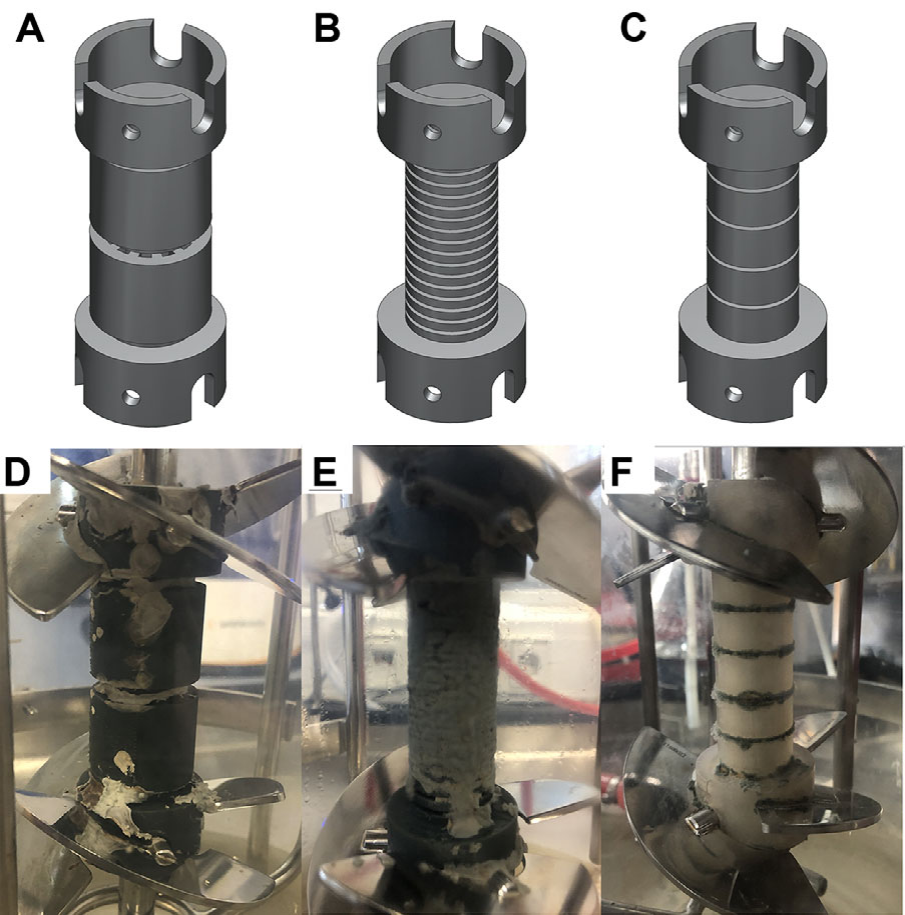
CAD drawings (top row) and photos after sporulation (bottom row) of different types of spinning sporulation support made of HTM 140 V.2 (A and D = type S1, B and E = type S2a) and E‐Shell 600 clear (C and F = type S2b)

The spinning sporulation support was designed and developed to be mounted on the stirrer shaft between the two stirrers. This arrangement should minimize the interference of the flow in the bioreactor. Because the spinning sporulation support acts like a thicker stirrer shaft, it should cause less additional shear forces than the fixed sporulation support. The rotation of the sporulation support should also prevent the deposition of excessive amounts of biomass on the outside of this sporulation support compared to the fixed sporulation support. The deposition should only occur in protected areas because of the shear forces on the flat rotating outer surface. Two types of spinning sporulation supports were designed (see Figure 2): type S1 with an outer closed structure and type S2 with grooves on the outside, similar to those of the fixed sporulation support type F1. Type S1 protected fungal growth in vertical slots.

Fermentation with sporulation support type S1 resulted in the desired growth in pellet form and a BDM of 5.09 ± 0.55 g/L at the end of fermentation (first batch phase). Although biomass deposition on the support was very low (see Figure 2A), a spore concentration of 3.75 × 10^7^ spores/L, according to Section 2.4, was achieved after the subsequent sporulation phase. This value was more than sufficient for reactor inoculation. In contrast, fermentation with type S2a showed a lower BDM of 3.88 ± 0.31 g/L. More biomass was deposited on the sporulation support (see Figure 2B). Because type S2a was equipped with grooves, a larger surface area was provided for the colonization of the fungus compared to type S1. As a result, the spore concentration with type S2a was 9.00 × 10^8^ spores/L, which was 24‐times higher than that of type S1.

Further optimization of the sporulation support type S2 was conducted by providing fewer grooves (type S2b). In addition, instead of HTM 140 V.2 (dark gray), the E‐Shell 600 clear (colorless‐transparent) material was used in 3D printing. Both measures could improve biomass deposition in the desired way (see Figure 2F). Biomass accumulated only in the grooves of sporulation support. The cross‐flow during agitation and the smooth material E‐Shell 600 clear prevented the accumulation of further biomass on the surface as desired. The BDM at the end of fermentation (first batch phase) was 5.72 ± 0.42 g/L, which was the best result achieved with the spinning sporulation supports.

After the sporulation step, a sufficient spore concentration of 3.76 × 10^7^ spores/L was achieved. The subsequent fermentation (second batch phase) with *Penicillium* sp. (IBWF 040‐09) showed a typical pH course and normal metabolic activity. A shortcoming of the spinning sporulation support is the positioning between the stirrers, which is not possible in every case.

### 3.3 Measurement of the fermentation progress with a backscattered light sensor

Tests were conducted to determine whether online process monitoring was possible with the non‐invasive sensor CGQ BioR (Aquila Biolabs GmbH and Infors AG). It measures the backscattered light through the reactor wall (for laboratory glass reactors). Higher cell densities increase the probability of backscattering. Therefore, the backscattered light is theoretically proportional to the cell concentration. This principle works very well in the fermentation of bacteria and yeast. [31, 32, 33]. In contrast, Jansen et al. [34] reported problems regarding the reproducibility of measuring fungi that grew in pellet form in microbioreactors. Nevertheless, this study determined whether the sensor was suitable for application in the present case. This sensor was advantageous because it was attached to the outside of the bioreactor wall; thus, it did not generate additional shear forces in the bioreactor.

**FIGURE 3:**
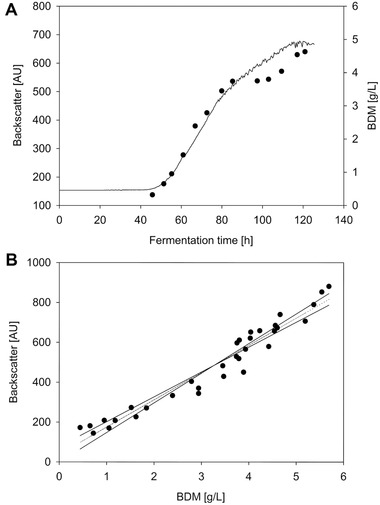
Online data from CGQ BioR sensor (A, line) and offline data from bio dry mass determination (A, dots) and correlation between bio dry mass and sensor signal in exponential phase (B)

The sensor could detect the increase in biomass concentration in the exponential phase, as shown by the comparison with the BDM determined in parallel (see Figure 3A). Using measurements in triplicate and a statistical assessment (*t‐*test), which was previously described [7], a good correlation occurred between the sensor signal and the BDM with 95 % prediction accuracy (see Figure 3B). The linear correlation was 136.72 ± 11.97 AU × L/g for *Penicillium* sp. (IBWF 040‐09) fermented under the experimental parameters presented here. The resulting deviation was mainly because the biomass was also deposited on the reactor wall in the vicinity of the sensor position distorting the measurement results. However, as is shown in Section 3.4, this biomass could be removed between batch phases with intermediate cleaning. Therefore, CGQ BioR is a suitable sensor for automating fungal fermentation in pellet form in conjunction with a repeated‐batch process.

### 3.4 Cleaning lance

The main idea was to determine a suitable approach for long‐term fermentation in pellet form to produce a sufficient amount of the target substance for further studies of pharmaceutical activity. This process should be feasible without “dead times” for cleaning and sterilization and without additional work to produce spore suspensions for inoculation. A cleaning phase between batch phases is an important step to ensure growth in pellet form and the functionality of the pH‐ and pO_2_‐sensors. Therefore, a cleaning device that could be implemented in repeated‐batch fermentation was also introduced and tested in this study. In addition, biofouling, which could occur during fermentation even with the growth in pellet form, will lead to an inaccurate measurement of biomass concentration, both online and offline. In the case of the online measurement presented in Section 3.3, any biofouling on the bioreactor wall will also result in an incorrect interpretation of the sensor signal using a calibration curve because the calibration curve was made in the absence of biofouling. This is one reason why cleaning devices are necessary.

**FIGURE 4:**
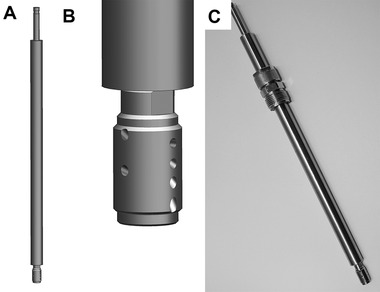
CAD drawings of the cleaning lance (A) and self‐rotating nozzle PicoWhirly (B) and photo of the cleaning lance with a compression fitting (C)

Fixed or rotating nozzle systems are implemented in cleaning industrial reactors and bioreactors. However, no cleaning nozzle system is commercially available for laboratory bioreactors. Therefore, a “cleaning lance” with a self‐rotating nozzle that could be installed in a PG 13.5 port or with an adapter in an M26 × 1 port was designed and built (see Figure 4). Height adjustment was possible by compression fitting. The nozzle PicoWhirly (Lechler GmbH) was selected as a suitable nozzle for the existing 2–10 L bioreactors.

**FIGURE 5:**
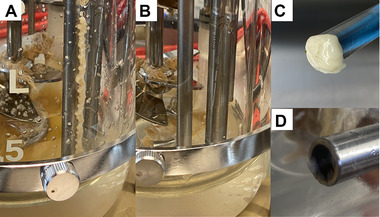
Biofouling after a batch fermentation of 140 h (A) and cleaning effect of the cleaning lance (B); dissolved oxygen sensor before (C) and after cleaning with the cleaning lance (D)

Commonly used alkalis, acids, or cleaning solutions cannot be used in the repeated‐batch process because they prevent sporulation. Therefore, we investigated whether a sufficient cleaning effect could also be achieved when operating the rotary nozzle with sterilized water. STT sterile coupling (Sartorius Stedim Biotech GmbH) was installed on the hose supply line of the cleaning lance. This allowed the bioreactor to be autoclaved as usual. The sterilized water required to operate the cleaning lance was autoclaved separately in a pressure vessel with a riser tube (pressure cask). The corresponding counterpart of sterile coupling was mounted on the riser tube. A sterile filter was placed at the compressed air connection of the pressure cask. The complete assembly was sterilized in an autoclave. After connecting the sterile coupling, it was possible to rinse the bioreactor. The maximum permissible pressure of the first test setup was 2 bar_g_. At this pressure and with a suitably selected position of the cleaning lance, a reasonably good cleaning effect was achieved (see Figure 5). In particular, we wanted to ensure growth in pellet form and to maintain the functionality of the pH‐ and pO_2_‐sensors. Both are already possible with the cleaning performance achieved so far. However, because (small) biomass residues are present, further optimization with regard to position and pressure must be conducted to achieve a cleaning effect at the desired level.

Because of the geometry of the sporulation support (see Section 3.2), part of the biomass remained on the sporulation support in the bioreactor as desired, despite the use of the cleaning lance. Moreover, the remaining biomass could sporulate after cleaning. The cleaning lance and pressure cask could also be used to refill the bioreactor with fresh medium, which allowed the spores that form on the sporulation support to be better rinsed and uniformly suspended in the fermentation medium. Sterilization of the medium in an autoclave could be performed using a pressure cask.

### 3.5 Repeated‐batch fermentation

After all individual steps for the planned repeated‐batch process were successfully implemented (see Sections 3.2, 3.3, and 3.4), it was investigated whether the desired process flow could be carried out. In this process, the spores formed on the biomass retained by the sporulation support were rinsed off and suspended via the cleaning lance at the beginning of the following batch phase, when the bioreactor was refilled with fresh medium. A problem caused by aeration during sporulation was identified. Some of the spores formed on the sporulation support were already distributed in the reactor during the sporulation phase if the airflow rate was too high. This led to an undesirable high deposition of biomass on the internals and in the headspace of the bioreactor. Therefore, the airflow rate must be reduced in the sporulation phase to 0.1 vvm, which is sufficient.

**FIGURE 6:**
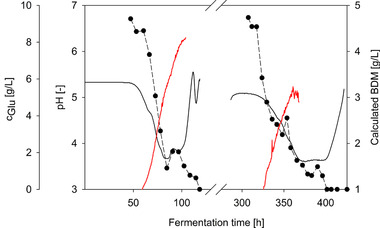
Course of the pH value (black line), glucose concentration (dots), and the biomass concentration calculated from the signal of the backscatter sensor (red line) in a repeated‐batch fermentation; starting at 370 h, the backscatter signal was interfered with by the biofouling on the bioreactor wall

Figure 6 shows the typical pH curve and glucose consumption profile for the fermentation of *Penicillium* sp. (IBWF 040‐09) in both phases of repeated‐batch fermentation. The biomass growth calculated from the values of the backscattered light sensor also showed a similar trend of fungal growth in both batch phases. However, the signal of the backscatter sensor was interfered with starting from t = 370 h, which is because the cleaning lance was not yet optimized to ensure proper intermediate cleaning of the bioreactor. During the subsequent batch phase, accumulation of new mycelia was observed on the remaining biomass, which was located in the upper part of the bioreactor wall and some parts of the stirrer (see Figure 4). The accumulation of biomass on the reactor wall in the area of the backscatter sensor led to measurement errors. Further optimization of the cleaning process is expected to significantly improve the online measurement of growth in this case.

## 4 CONCLUDING REMARKS

An automated long‐term fermentation process of the filamentous fungus *Penicillium* sp. (IBWF 040‐09) in pellet form was designed and investigated in this study. Sporulation support, cleaning lance, and backscattered CGQ sensors were implemented in repeated‐batch fermentation. The purpose of the sporulation support in repeated‐batch fermentations was to accumulate a suitable amount of biomass, keep this in the bioreactor during intermediate cleaning, and enable a subsequent sporulation phase. The spores were then used as inocula for the next batch phase. This approach ensured fungal growth in the form of pellets. The amount of biomass retained by the sporulation supports was adjusted by changing the geometry. The interaction between the designs of the sporulation supports on the one hand, and the intermediate cleaning on the other is important. Using suitable geometries, we have achieved different amounts of biomass formed on the sporulation supports, which are rinsed off during intermediate cleaning in such a way that the remaining biomass produces a spore concentration that is within the desired range of values. The complete procedure for repeated‐batch fermentation is summarized as follows:

Step 1: First batch phase at 22°C with spore suspension as the inoculum.
Step 2: Determination of fungal growth with the CGQ sensor (see Section 3.3); stop the batch phase when the sensor signal indicates a decreasing growth rate or when the aeration rate decreases to 1 L/min.
Step 3: Harvest the biomass and empty the bioreactor (preferably via a bottom drain valve).
Step 4: Intermediate cleaning of the bioreactor with a cleaning lance by rinsing with sterilized water (see Section 3.4).
Step 5: Starting the sporulation phase with the help of sporulation support (see Section 3.2) at 22°C with humid aeration (see Section 3.1) and reduced airflow rate (see Section 3.5).
Step 6: Refilling the bioreactor with fresh medium via cleaning lance and uniform suspension of spores in the medium.
Step 7: Start the next batch phase by increasing the airflow.
Step 8: Continue with Step 2

With this concept, biofouling on the surfaces of the bioreactor and sensors can be avoided and removed during the process. Consequently, the pellet form could be maintained in repeated‐batch fermentation. To evaluate the fermentation strategy with the sporulation support in more detail, the fungal morphology and the production of the target substance are being investigated in studies already underway.

## CONFLICT OF INTEREST

The authors declare no conflict of interest.

## Data Availability

The data that support the findings of this study are available from the corresponding author upon reasonable request.

## References

[1] Aharonowitz Y , Cohen G , Martin JF . Penicillin and cephalosporin biosynthetic genes: structure, organization, regulation, and evolution. Annu Rev Microbiol. 1992; 46: 461‐495. 1444264 10.1146/annurev.mi.46.100192.002333

[2] Zou SP , Xiong Y , Niu K , Hu ZC , Zheng YG , Integrated strategy of temperature shift and mannitol feeding for enhanced production of echinocandin B by Aspergillus nidulans CCTCC M2012300. 3 Biotech 2019, 9, 140. 10.1007/s13205-019-1668-x PMC6419687 30944787

[3] Feofilova EP . Karotinoidy gribov: biologicheskie funktsii i prakticheskoe ispol'zovan [Fungal carotenoids: biological functions and practical use]. Prikl Biokhim Mikrobiol. 1994; 30: 181‐195. 8183855

[4] De Carli L , Larizza L . Griseofulvin. Mutat Res. 1988;195:91‐126. 3277037 10.1016/0165-1110(88)90020-6

[5] Kato T , Park EY . Riboflavin production by Ashbya gossypii. Biotechnol Lett. 2012; 34: 611‐618. 22187081 10.1007/s10529-011-0833-z

[6] Ettari R , Previti S , Tamborini L , et al. The inhibition of cysteine proteases rhodesain and TbCaTB: a valuable approach to treat human African trypanosomiasis. Mini Rev Med Chem. 2016; 16: 1374‐1391. 27156518 10.2174/1389557515666160509125243

[7] Soerjawinata W , Schlegel K , Fuchs N , et al. Applicability of a single‐use bioreactor compared to a glass bioreactor for the fermentation of filamentous fungi and evaluation of the reproducibility of growth in pellet form. Eng Life Sci. 2021; 21: 324‐339. 33976605 10.1002/elsc.202000069 PMC8092982

[8] Krull R , Wucherpfennig T , Esfandabadi ME , et al. Characterization and control of fungal morphology for improved production performance in biotechnology. J Biotechnol. 2013; 163: 112‐123. 22771505 10.1016/j.jbiotec.2012.06.024

[9] Grimm LH , Kelly S , Krull R , Hempel DC . Morphology and productivity of filamentous fungi. Appl Microbiol Biotechnol. 2005; 69: 375‐384. 16317480 10.1007/s00253-005-0213-5

[10] Posch AE , Herwig C , Spadiut O . Science‐based bioprocess design for filamentous fungi. Trends Biotechnol. 2013; 31: 37‐44. 23183302 10.1016/j.tibtech.2012.10.008

[11] Veiter L , Kager J , Herwig C . Optimal process design space to ensure maximum viability and productivity in Penicillium chrysogenum pellets during fed‐batch cultivations through morphological and physiological control. Microb Cell Fact. 2020;19:33. 32054538 10.1186/s12934-020-1288-5 PMC7020361

[12] Gbewonyo K , Wang DIC . Enhancing gas‐liquid mass transfer rates in non‐Newtonian fermentations by confining mycelial growth to microbeads in a bubble column. Biotechnol Bioeng. 1983; 25: 2873‐2887. 18548624 10.1002/bit.260251206

[13] Zhang J , Zhang J . The filamentous fungal pellet and forces driving its formation. Crit Rev Biotechnol. 2016; 36: 1066‐1077. 26381130 10.3109/07388551.2015.1084262

[14] Takahashi J , Yamada KJ . Studies on the effects of some physical conditions on the submerged mold culture. Part III: relations between the morphological forms of molds and the viscosity of mycelial suspensions. J Agric Chem Soc Jpn. 1960; 34: 100‐103.

[15] Chain EB , Gualandi G , Morisi G . Aeration studies IV. Aeration conditions in 3000‐liter submerged fermentations with various microorganisms. Biotechnol Bioeng. 1966; 8: 595‐619.

[16] Meyer V , Cairns T , Barthel L , et al. Understanding and controlling filamentous growth of fungal cell factories: novel tools and opportunities for targeted morphology engineering. Fungal Biol Biotechnol. 2021; 8: 8. 34425914 10.1186/s40694-021-00115-6 PMC8383395

[17] Gbewonyo K , Hunt G , Buckland B . Interactions of cell morphology and transport processes in the lovastatin fermentation. Bioprocess Eng. 1992; 8: 1‐7.

[18] Hille A , Neu TR , Hempel DC , Horn H . Einfluss der Morphologie auf Stofftransport und ‐Umsatz in Aspergillus niger‐pellets. Chem Ing Tech. 2006; 78: 627‐632.

[19] Posch AE , Spadiut O , Herwig C . A novel method for fast and statistically verified morphological characterization of filamentous fungi. Fungal Genet Biol. 2012; 49: 499‐510. 22587949 10.1016/j.fgb.2012.05.003

[20] Cronenberg CCH , Ottengrad SPP , van den Heuvel JC , et al. Influence of age and structure of Penicillium chrysogenum pellets of the internal concentration profiles. Bioproc Eng. 1994; 10: 209‐216.

[21] Vanhoutte B , Pons MN , Thomas CR , Louvel L , Vivier H . Characterization of Penicillium chrysogenum physiology in submerged cultures by color and monochrome image analysis. Biotechnol Bioeng. 1995; 48: 1‐11. 18623454 10.1002/bit.260480103

[22] White S , McIntyre M , Berry DR , McNeil B . The autolysis of industrial filamentous fungi. Crit Rev Biotechnol. 2002; 22: 1‐14. 11958333 10.1080/07388550290789432

[23] Smith JJ , Lilly MD , Fox RI . The effect of agitation on the morphology and penicillin production of Penicillium chrysogenum. Biotechnol Bioeng. 1990; 35: 1011‐1023. 18588247 10.1002/bit.260351009

[24] Li G , Chen Z , Chen N , Xu Q . Enhancing the efficiency of L‐tyrosine by repeated batch fermentation. Bioengineered. 2020; 11: 852‐861. 32749196 10.1080/21655979.2020.1804177 PMC8291887

[25] Wan‐Mohtar WAAQI , Ab Kadir SA , Saari N . The morphology of Ganoderma lucidum mycelium in a repeated‐batch fermentation for exopolysaccharide production. Biotechnol Rep (Amst). 2016; 11: 2‐11. 28352534 10.1016/j.btre.2016.05.005 PMC5042302

[26] Yang X , Wang B , Cui F , Tan T . Production of lipase by repeated batch fermentation with immobilized Rhizopus arrhizus. Process Biochem. 2005; 40 (6):2095‐2103.

[27] Driouch H , Sommer B , Wittmann C . Morphology engineering of Aspergillus niger for improved enzyme production. Biotechnol Bioeng. 2010; 105: 1058‐1068. 19953678 10.1002/bit.22614

[28] Grijseels S , Nielsen JC , Nielsen J , et al. Physiological characterization of secondary metabolite producing Penicillium cell factories. Fungal Biol Biotechnol. 2017; 4: 8. 29075506 10.1186/s40694-017-0036-z PMC5644182

[29] Pera LM , Callieri DA . Influence of calcium on fungal growth, hyphal morphology and citric acid production in Aspergillus niger. Folia Microbiol. 1997; 42: 551‐556. 9438355 10.1007/BF02815463

[30] Sieblist C , Jenzsch M , Pohlscheidt M . Influence of pluronic F68 on oxygen mass transfer. Biotechnol Prog. 2013; 29: 1278‐1288. 23843368 10.1002/btpr.1770

[31] Halmschlag B , Putri SP , Fukusaki E , Blank LM . Poly‐γ‐glutamic acid production by Bacillus subtilis 168 using glucose as the sole carbon source: a metabolomic analysis. J Biosci Bioeng. 2020; 130: 272‐282. 32546403 10.1016/j.jbiosc.2020.04.011

[32] Hitschler J , Boles E . De novo production of aromatic m‐cresol in Saccharomyces cerevisiae mediated by heterologous polyketide synthases combined with a 6‐methylsalicylic acid decarboxylase. Metab Eng Commun. 2019; 9: e00093. 31193192 10.1016/j.mec.2019.e00093 PMC6520567

[33] Sörensen M , Khakimov B , Nurjadi D , et al. Comparative evaluation of the effect of different growth media on in vitro sensitivity to azithromycin in multi‐drug resistant Pseudomonas aeruginosa isolated from cystic fibrosis patients. Antimicrob Resist Infect Control. 2020; 9: 197. 33298147 10.1186/s13756-020-00859-7 PMC7724801

[34] Jansen RP , Beuck C , Moch M , et al. A closer look at Aspergillus: online monitoring via scattered light enables reproducible phenotyping. Fungal Biol Biotechnol. 2019; 6: 11. 31396392 10.1186/s40694-019-0073-x PMC6681481

